# Co-Occurrence of Language and Behavioural Change in Frontotemporal Lobar Degeneration

**DOI:** 10.1159/000444848

**Published:** 2016-06-01

**Authors:** Jennifer M. Harris, Matthew Jones, Claire Gall, Anna M.T. Richardson, David Neary, Daniel du Plessis, Piyali Pal, David M.A. Mann, Julie S. Snowden, Jennifer C. Thompson

**Affiliations:** ^a^Manchester Academic Health Sciences Centre, Cerebral Function Unit, Greater Manchester Neuroscience Centre, Salford Royal NHS Foundation Trust, Salford, Manchester, UK; ^b^Institute of Brain, Behaviour and Mental Health, University of Manchester, Manchester, UK

**Keywords:** Frontotemporal dementia, Frontotemporal lobar degeneration, Aphasia, Pathology, Diagnostic criteria

## Abstract

**Background/Objectives:**

We aimed to evaluate the co-occurrence of language and behavioural impairment in patients with frontotemporal lobar degeneration (FTLD) spectrum pathology.

**Methods:**

Eighty-one dementia patients with pathological confirmation of FTLD were identified. Anonymized clinical records from patients' first assessment were rated for language and behavioural features from frontotemporal dementia consensus criteria, primary progressive aphasia (PPA) criteria and 1998 FTLD criteria.

**Results:**

Over 90% of patients with FTLD pathology exhibited a combination of at least one behavioural and one language feature. Changes in language, in particular, were commonly accompanied by behavioural change. Notably, the majority of patients who displayed language features characteristic of semantic variant PPA exhibited ‘early perseverative, stereotyped or compulsive/ritualistic behaviour’. Moreover, ‘executive/generation deficits with relative sparing of memory and visuospatial functions’ occurred in most patients with core features of non-fluent variant PPA.

**Conclusion:**

Behavioural and language symptoms frequently co-occur in patients with FTLD pathology. Current classifications, which separate behavioural and language syndromes, do not reflect this co-occurrence.

## Introduction

Three main clinical dementia syndromes are associated with frontotemporal lobar degeneration (FTLD) pathology. These are characterized by predominant changes in behaviour, expressive language and semantic knowledge. Currently the behavioural disorder, behavioural variant frontotemporal dementia (bvFTD), is encapsulated by 2011 FTD consensus criteria [1], whereas non-fluent and semantic syndromes are incorporated within distinct primary progressive aphasia (PPA) classifications [2]. Therefore, linguistic and behavioural features of FTLD may be evaluated separately and their co-occurrence overlooked.

In a previous study, we found that some patients presenting with prominent language impairment also had prominent behavioural problems, which was associated with FTLD pathology [3]. Other studies have reported behavioural changes in non-fluent and semantic variant PPA (nfvPPA and svPPA, respectively) [4,5,6,7], and it has been argued that such behavioural changes may aid differentiation of PPA due to FTLD spectrum pathology from PPA due to Alzheimer's disease pathology [8]. Certain language characteristics have been reported in bvFTD, in particular poor organization of speech, echolalia and perseveration [9,10,11,12]. These features are often considered in terms of frontal executive dysfunction; however, core features of svPPA and nfvPPA have rarely been investigated in bvFTD. The co-occurrence of characteristic features of the three main clinical manifestations of FTLD has not yet been explored in pathologically confirmed FTLD.

The aim of this study was to evaluate the co-occurrence of language and behavioural features in patients with FTLD pathology. We hypothesized that language and behavioural features frequently co-exist in patients with FTLD pathology.

## Materials and Methods

### Participants

**Fig. 1:**
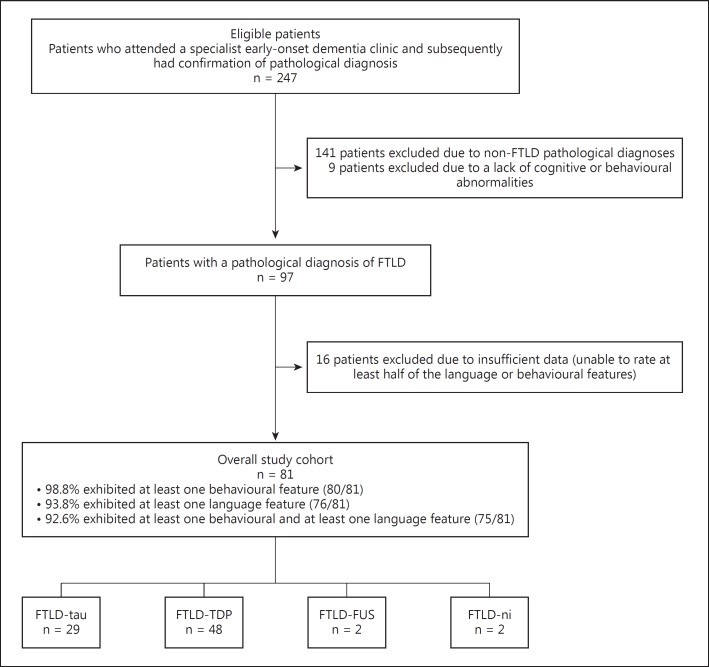
Case selection.

Participants were selected from a cohort of 247 patients who had attended a specialist early-onset dementia clinic and had pathological confirmation of diagnosis. All brains were acquired through an ethically approved study with informed written consent. The selection of cases is shown in figure 1, the criteria for selection being the presence of FTLD pathology together with any cognitive or behavioural impairment (n = 97). In order to reduce missing data, patients were excluded if it was impossible to rate at least 50% of the language and/or behavioural features. The final cohort comprised 81 patients (50 male and 31 female), whose mean age at onset of symptoms was 58.1 years (SD 9.6 years) and mean duration of symptoms at referral was 3.2 years (SD 3.0 years). Thirty-six patients (45%) (2 missing) had a family history of a similar disorder in a first-degree relative.

The FTLD pathologies of the 81 patients, classified in accordance with current pathological criteria [13,14] were as follows: 29 FTLD-tau (1 progressive supranuclear palsy, 9 corticobasal degeneration, 9 FTDP-17Tau, 10 Pick's disease), 48 FTLD TDP-43 (24 type A, 17 type B and 7 type C), 2 no inclusions and 2 fused in sarcoma. The cohort encompassed a range of clinical diagnoses, including patients who had been clinically classified as having ‘mixed’ presentations. Forty-seven patients presented with an FTD syndrome, 11 of whom exhibited additional semantic impairment. Eight patients presented with a circumscribed semantic impairment and 13 were clinically classified as having progressive non-fluent aphasia. Ten patients presented with FTD with motor neurone disease, 1 with corticobasal syndrome and 2 with progressive supranuclear palsy syndrome.

Genetic screening for progranulin (PGRN), microtubule associated protein tau (MAPT) and C9orf72 was available for 56/81 (71.6%) patients. Six patients had a C9orf72 expansion, 8 had PGRN mutations and 9 had MAPT mutations.

### Materials

Clinic letters and reports from patients' initial visit were copied and personal identifiable and diagnostic information removed. The available data included a comprehensive cognitive and behavioural history, detailed neurological examination, reports from neuroimaging, other investigations and neuropsychological assessment. The cognitive/behavioural history, taken from a close informant, was obtained using a standardized semi-structured interview that includes questions relating to language (expression, comprehension, naming, reading and writing), executive skills (attention, reason and judgement, planning and organization) and behaviour and affect (sympathy, empathy, depression, anxiety, irritability, apathy, disinhibition, dietary change, repetitive behaviours and stereotypies). Cognitive assessment incorporated, for all patients, the Manchester Neuropsychological Profile (MNP) [15,16], an assessment tool that taps different domains of cognition and has been shown to be useful in distinguishing between different forms of dementia [15]. Where a deficit in a specific domain was detected on the MNP (e.g. semantic impairment), additional tests had typically also been undertaken, which provided confirmatory evidence of the abnormality. Data on these supplementary tests were not available for all patients. The MNP's evaluation of language is described in detail elsewhere [3] and includes analysis of form and content of conversational speech, speech and buccofacial praxis, phonemic and semantic errors, single word and sentence comprehension, digit, word and phrase repetition, naming, reading, writing and spelling. The MNP taps executive skills, including organization and sequencing (verbal, pictorial and motor), abstraction (proverb interpretation), set shifting (Weigl's blocks) and generation (category and letter fluency). Fluency deficits were interpreted as evidence of executive impairment only if they were disproportionate to deficits in confrontation naming. The MNP records qualitative aspects of patients' behaviour during testing including impulsivity, distractibility, economy of effort, rule violations and perseverations.

### Procedure

Redacted documents were inspected for the presence or absence of behavioural and linguistic features by one of two raters who had not been involved in the patients' clinical care and were blind to clinical diagnosis and underlying pathology. In order to reduce false negative attributions, features that were not explicitly documented were rated as ‘missing’ rather than absent. The behavioural and language features were drawn from current consensus criteria for bvFTD [1] and PPA (including core features of svPPA, nfvPPA and logopenic variant PPA) [2], as well as the 1998 criteria for FTLD [17], which encompasses distinct but related criteria for the behavioural syndrome of FTD, semantic dementia and progressive non-fluent aphasia. The rationale for using criteria-based features is that these are critical for clinical characterization and differential diagnosis of FTLD syndromes. Information from patients' first evaluation including the detailed clinical history and neuropsychological reports based on the MNP was used to rate cognitive features. Cut-off scores from tests were not utilized since poor performance in one domain, such as language or executive function, can have a marked impact on test performance on all tests. Instead, ratings were made on the basis of the psychologist's description/interpretation of patients' performance on tests.

The behavioural features recorded comprised the core features from current consensus criteria for bvFTD [1]: early behavioural disinhibition, early apathy or inertia, early loss of sympathy or empathy, early perseverative, stereotyped or compulsive/ritualistic behaviour, hyperorality and dietary changes, as well as the cognitive feature, executive/generation deficits with relative sparing of memory and visuospatial functions.

The language features recorded included positive features from the PPA classifications [2] and some language features from the 1998 FTLD criteria [17]. Language features encompassed the following broad domains: speech production (effortful, halting speech with inconsistent speech sound errors and distortions), syntax (agrammatism in language production and impaired comprehension of complex syntax), phonology (phonemic errors and impaired repetition of sentences and phrases), semantics (impaired single-word comprehension, impaired object knowledge, surface dyslexia or dysgraphia, and semantic paraphasias) and frontal/executive (aspontaneity and economy of speech, echolalia, and perseveration in speech). In order to circumvent the issue as to whether these latter features should be considered as behavioural or language features, we considered them as language features likely predicated on frontal/executive dysfunction. Some additional features could conceivably be due to impairments in more than one domain (phonology/semantics: impaired confrontation naming; semantics/frontal: stereotypy of speech and idiosyncratic word usage).

### Statistical Analysis

Data analysis was carried out using IBM SPSS statistics version 20.

## Results

There was a large degree of overlap between language and behavioural features in patients with FTLD pathology. Seventy-five (of 81) patients displayed a combination of at least one bvFTD feature and at least one language feature. Five patients exhibited at least one bvFTD feature but no language features. One patient exhibited at least one language feature but no features of bvFTD.

**Fig. 2:**
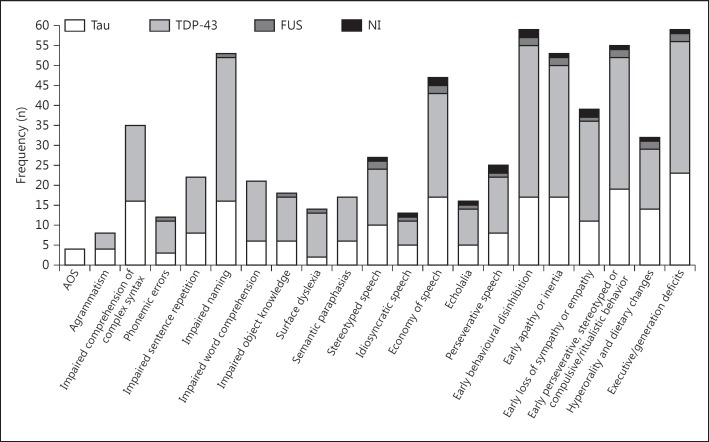
Frequency of language and behavioural symptoms and pathological relationships. The frequencies shown are for a maximum of 81 patients: 29 FTLD-tau (Tau), 48 FTLD TDP-43 (TDP-43), 2 no inclusions (NI) and 2 fused in sarcoma (FUS).

The frequency of individual behavioural and language features is shown in figure 2. Behavioural features were more common than language features within the cohort, and most features occurred in a similar proportion of TDP-43 and tau pathologies. There was one notable exception, apraxia of speech (AOS) occurred only in patients with tau pathology.

Notably, a high proportion of FTLD patients who exhibited ‘impaired word comprehension’ and ‘impaired object knowledge’, features which are characteristic of svPPA, also exhibited ‘early perseverative, stereotyped or compulsive/ritualistic behaviour’ (18/21 and 16/18 respectively; table 1). ‘Executive/generation deficits with relative sparing of memory and visuospatial functions’ were common in patients who exhibited ‘AOS’ or ‘agrammatism’, which are core features of nfvPPA (3/4 and 6/8 respectively; table 1).

## Discussion

The study found that language and behavioural features frequently co-occur in patients with FTLD pathology. In particular, the presence of behavioural features was common in patients who exhibited language symptoms.

### Behavioural Change in Patients with Language Impairment

The presence and type of behavioural features was not uniform across patients who exhibited core language features. The majority of patients who displayed language features characteristic of svPPA exhibited ‘early perseverative, stereotyped or compulsive/ritualistic behaviour’. In contrast, this feature occurred less frequently in patients who exhibited core features of nfvPPA. Other studies have reported similar behavioural changes in semantic dementia/svPPA [5,6,7,18,19]. The association between this behavioural feature and those language features that are characteristic of svPPA in patients with established FTLD pathology points to the usefulness of this feature for clinical diagnosis. The co-occurrence of behavioural abnormalities and semantic deficits raises questions over the classification of svPPA as a primary aphasic disorder.

Behavioural features were common too in FTLD patients who exhibited the core features of nfvPPA. Moreover, ‘executive/generation deficits with relative sparing of memory and visuospatial functions’ occurred in most patients. Impairments were elicited on non-verbal sorting and sequencing tasks that could not readily be ascribed to patients' aphasia. In view of the striking language disorders in nfvPPA, it is possible that non-verbal cognitive deficits may be overlooked or attributed to language impairment. Further research into non-verbal performance characteristics in nfvPPA is required, since the presence of executive impairment, even in the absence of overt behavioural change, may aid clinical diagnosis of nfvPPA.

### Language Impairment in Patients with Behavioural Change

Core features of PPA were relatively uncommon in FTLD patients who exhibited behavioural changes, particularly ‘AOS’ and ‘agrammatism’, core features of nfvPPA. However, some language and speech features did frequently occur. Most of these are typically associated with frontal-executive dysfunction and indeed were included in 1998 FTLD criteria for FTD [17]. Their co-occurrence with behavioural symptoms might therefore seem predictable. However, with the exception of ‘stereotypy of speech’ [1], these characteristic changes in speech and language are a notable omission from the FTDC criteria for bvFTD. Our data suggest that these features may prove useful diagnostically in the clinical setting. In addition, ‘impaired confrontation naming’ commonly occurred in the cohort and interestingly, other central features related to semantic dysfunction occurred in around a fifth of patients who exhibited behavioural change. This finding further highlights the links between semantic and behavioural dysfunction.

A recent longitudinal study suggested that language decline in bvFTD is similar to that observed in PPA [20]. Our findings suggest that at presentation the presence of language impairment is variable amongst patients with FTLD: some patients (n = 5) exhibited no language features, whereas other patients exhibited core features of PPA subtypes together with behavioural change. It seems that a spectrum of disorders exists within patients with FTLD pathology, whereby patients can exhibit focal language disorders, focal behavioural disorders or a mixture of both.

### Criteria and Overlapping Language and Behavioural Disorders

The available evidence suggests that there is a degree of overlap between behavioural and linguistic symptoms associated with FTLD spectrum pathology. In particular, behavioural changes appear to be common in patients who exhibit core features of semantic and nfvPPA. This overlap is substantial; greater recognition of these commonly co-occurring features may aid clinical diagnosis. Yet, the separation of PPA classifications [2] from consensus criteria for bvFTD (FTDC criteria) [1] risks language and behavioural symptoms being evaluated independently. Moreover, ‘prominent initial behavioural disturbance’ is an exclusion criterion in basic PPA criteria [2]. Accurate clinical diagnosis according to underlying pathology is becoming increasingly reliant on biomarkers. Whilst this has great utility in the research setting, the expense is too high for current routine clinical work. The presence of common characteristics in bvFTD and PPA may improve clinico-pathological correlations. Further studies in larger cohorts with various pathologies are required to determine whether the co-occurrence of features of PPA and bvFTD can improve prediction of underlying pathology.

The study has limitations. Principally, albeit by necessity, it was carried out by retrospective blinded case note review, so rating of linguistic and behavioural features was dependent upon the information recorded at the time of patients' clinical evaluation. However, all patients were seen in a single centre and underwent a structured cognitive history and neuropsychological assessment protocol. Patients in whom there were insufficient clinical data were excluded from further analysis. All patients had attended a specialist early-onset dementia clinic with a focus on behavioural and language disorders. There is inherently referral bias for patients attending tertiary referral centres, and it is therefore possible that older-onset FTLD cases are underrepresented in our cohort; nevertheless, it is well established that FTLD is primarily a relatively young-onset disorder.

## Conclusions

In conclusion, behavioural and language symptoms commonly occur together in patients with underlying FTLD. There is a need for diagnostic criteria to reflect this co-occurrence and for further research to elucidate the way in which this can help predict FTLD versus non-FTLD pathology in vivo.

## Figures and Tables

**Table 1:** Frequency of co-occurring language and behavioural features in patients with FTLD pathology

	Early behavioural disinhibition (n = 59; 4 missing)	Early apathy or inertia (n = 53; 6 missing)	Early loss of sympathy or empathy (n = 39; 16 missing)	Early perseverative, stereotyped or compulsive/ritualistic behaviour (n = 55; 5 missing)	Hyperorality and dietary changes (n = 32; 28 missing)	Executive/generation deficits (n = 59; 13 missing)
AOS (n = 4; 2 missing)	1	1	1	1	1	3

Agrammatism (n = 8; 4 missing)	5	6	4	4	0	6

Impaired comprehension of complex syntax (n = 35; 22 missing)	24	25	20	23	10	32

Phonemic errors (n = 12; 4 missing)	9	7	4	6	4	7

Impaired sentence repetition (n = 22; 22 missing)	13	12	10	13	5	16

Impaired naming (n = 53; 2 missing)	41	30	26	39	22	36

Impaired word comprehension (n = 21; 3 missing)	19	10	11	18	11	13

Impaired object knowledge (n = 18; 6 missing)	15	8	10	16	11	10

Surface dyslexia (n = 14; 10 missing)	11	5	6	11	6	7

Semantic paraphasias (n = 17; 1 missing)	14	8	8	14	7	10

Stereotyped speech (n = 27; 5 missing)	21	15	17	27	15	21

Idiosyncratic speech (n = 13; 1 missing)	10	8	8	10	5	10

Economy of speech (n = 47; 0 missing)	35	35	25	36	21	36

Echolalia (n = 16; 0 missing)	14	11	13	14	10	13

Perseverative speech (n = 25; 3 missing)	21	19	16	21	10	16

n = Number of patients exhibiting each behavioural (column) and language (row) symptom; missing = number of patients with missing data for that particular feature.
